# Supplementary material for: The Role of Bcl-2 and Beclin-1 Complex in “Switching” between Apoptosis and Autophagy in Human Glioma Cells upon LY294002 and Sorafenib Treatment
Source: Cells. 2023 Nov 21;12(23):2670. doi: 10.3390/cells12232670 (PMC10705223; doi:10.3390/cells12232670)
Supplement: Supplementary file 1 [file cells-12-02670-s001.zip › cells-2709093-supplementary.pdf]

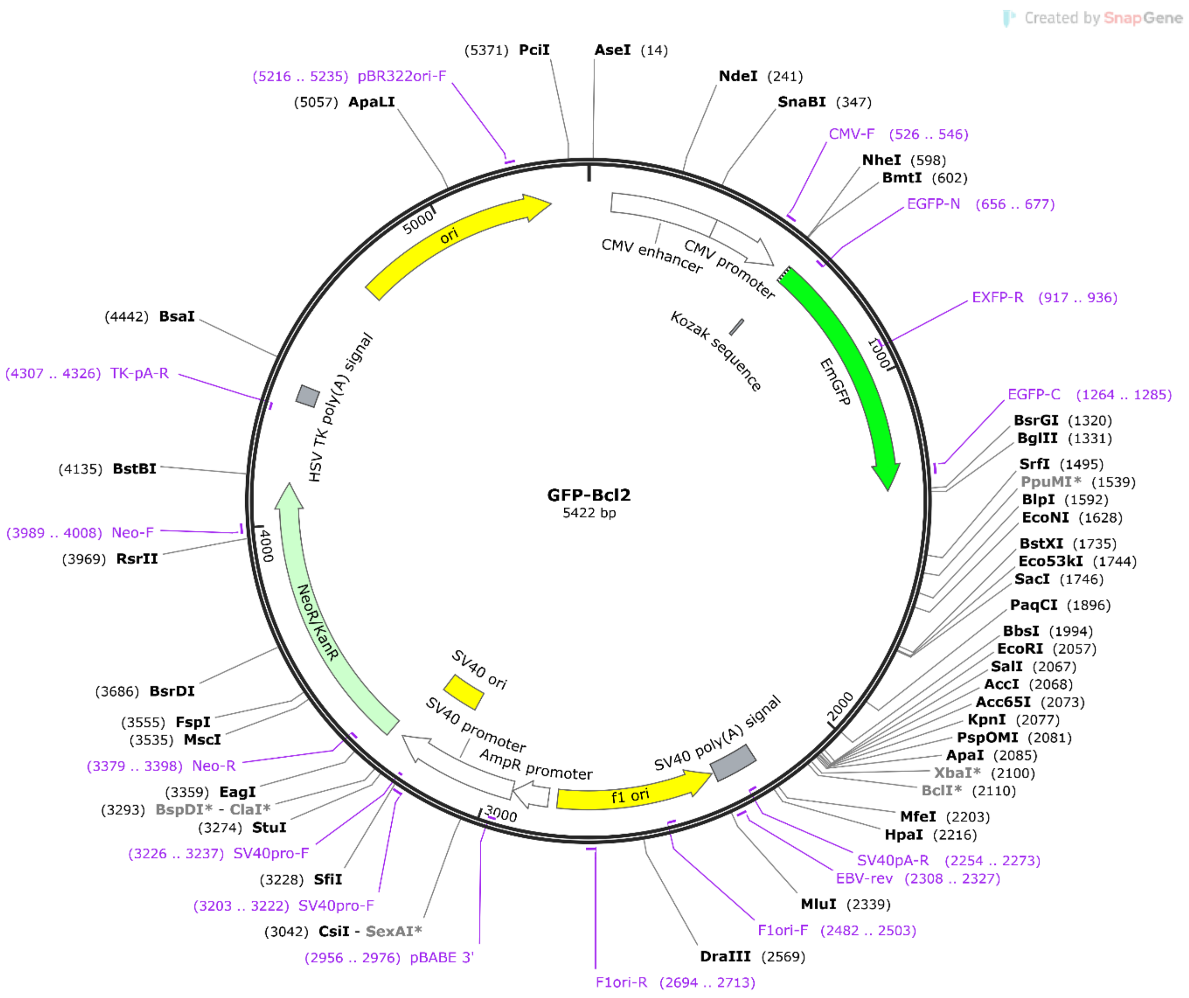

Fig. 1. Sequence map of GFP-Bcl2 plasmid used in experiments (AddGene, Watertown, USA).

Forward: 5'- AGA TCT CGA GCT ATG GAA GGC AGC - 3'
Reverse: 5' - GCT GGA TCC TTT GTT ATA AAA CTG - 3'

Fig. 2. Sequence of primers for cloning pEGFP-beclin-1 plasmid given from Cambridge University to pmCherryC-beclin-1 plasmid used in experiments.

A

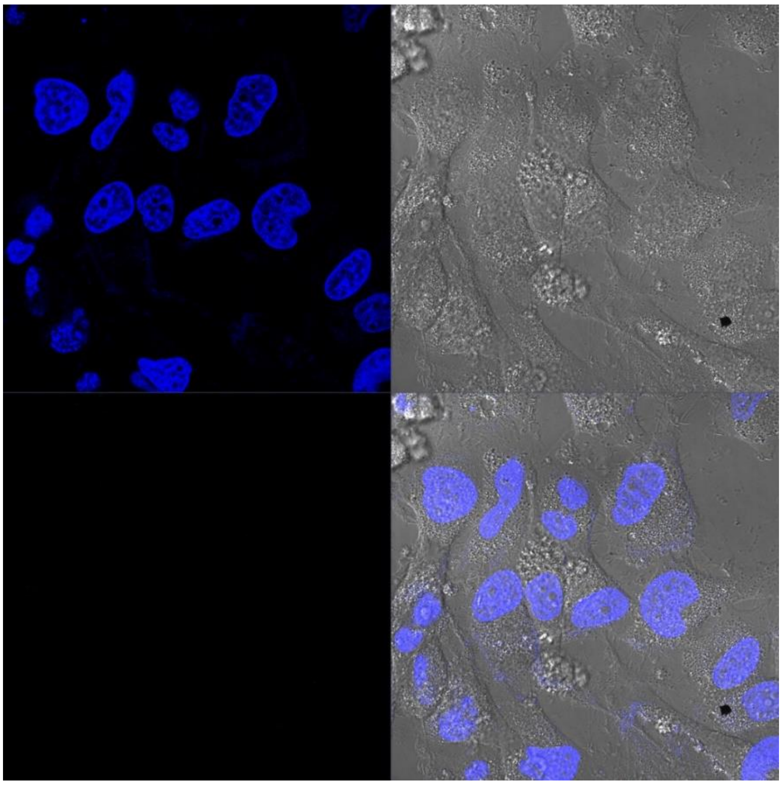

B

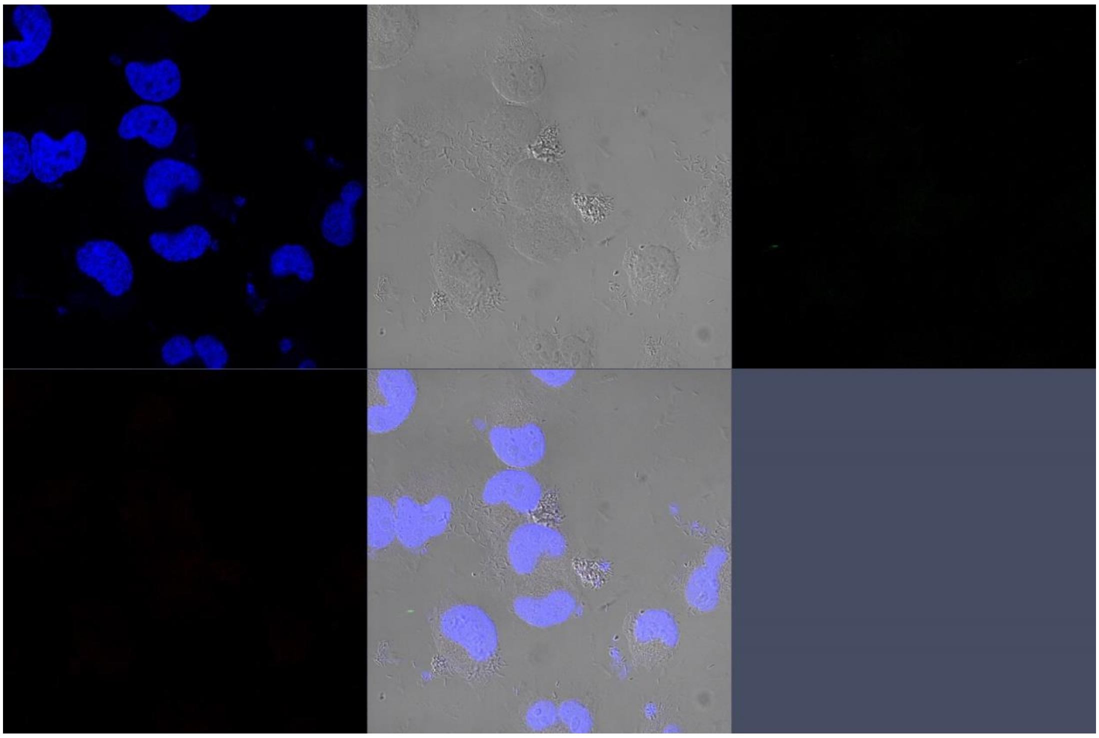

Fig. 3 Representative photo snapped with the Laser Scanning Confocal Microscopy system LSM780 Zeiss (Oberkochen, Germany) of Hoechst 33342 staining of **A**: Glioblastoma multiforme cells (T98G) treated with lipofectamine only in order to exclude its potential cytotoxicity and **B**: T98G untreated and untransfected cells as a control to exclude potentially autofluorescence.

A

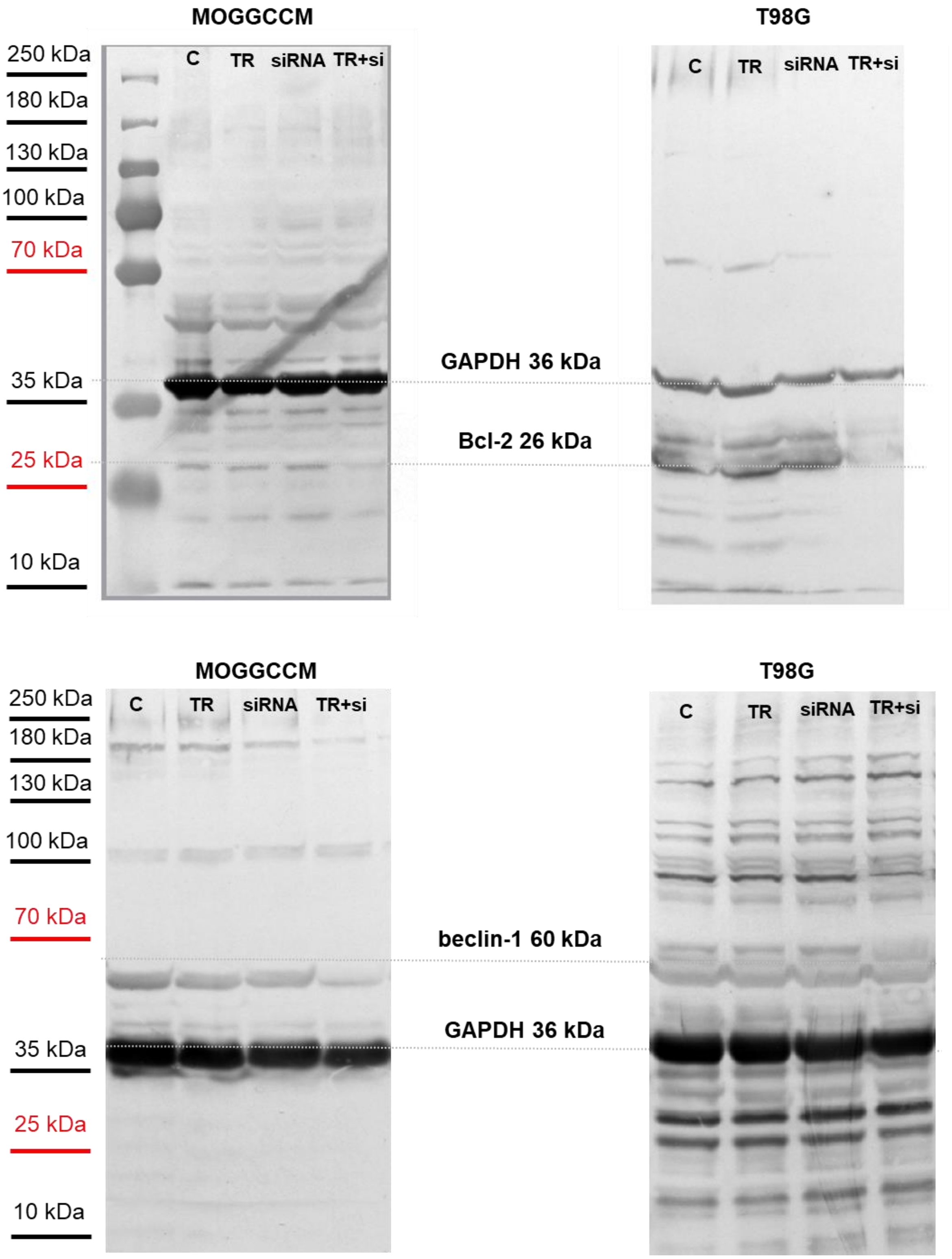
MOGGCCM
T98G
250 kDa
180 kDa
130 kDa
100 kDa
70 kDa
35 kDa
25 kDa
10 kDa
C
TR
siRNA
TR+si
GAPDH 36 kDa
Bcl-2 26 kDa
MOGGCCM
T98G
250 kDa
180 kDa
130 kDa
100 kDa
70 kDa
35 kDa
25 kDa
10 kDa
beclin-1 60 kDa
GAPDH 36 kDa

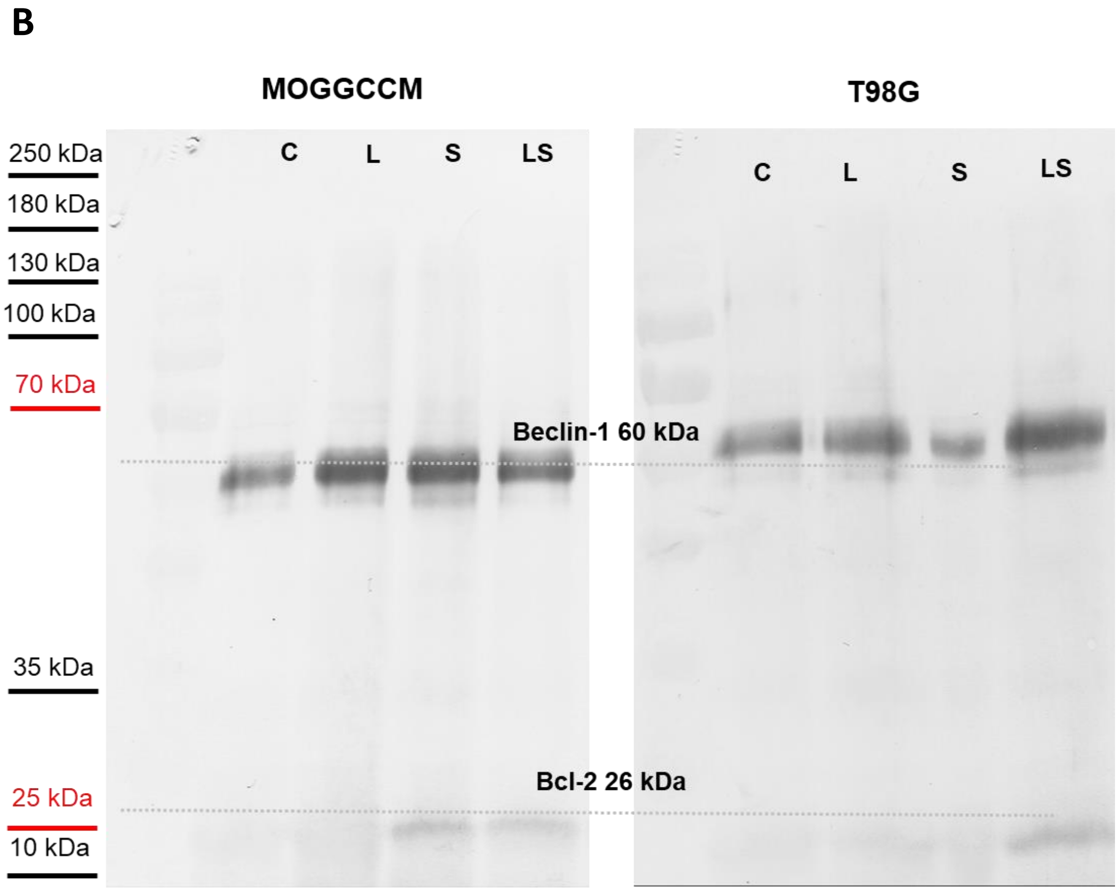

Fig. 4 A: Whole immunoblotting membranes showing the Bcl-2 and beclin-1 expression silencing in MOGGCCM and T98G transfected cells. C – control, TR – transfection reagent, siRNA – Bcl-2/beclin-1 expression silencer, TR+si – proper transfection mix; **B:** Whole immunoblotting membranes after co-immunoprecipitation assay showing the Bcl-2 and beclin-1 complex in MOGGCCM and T98G. C – control, L – LY294002, S – sorafenib, LS – LY294002+sorafenib

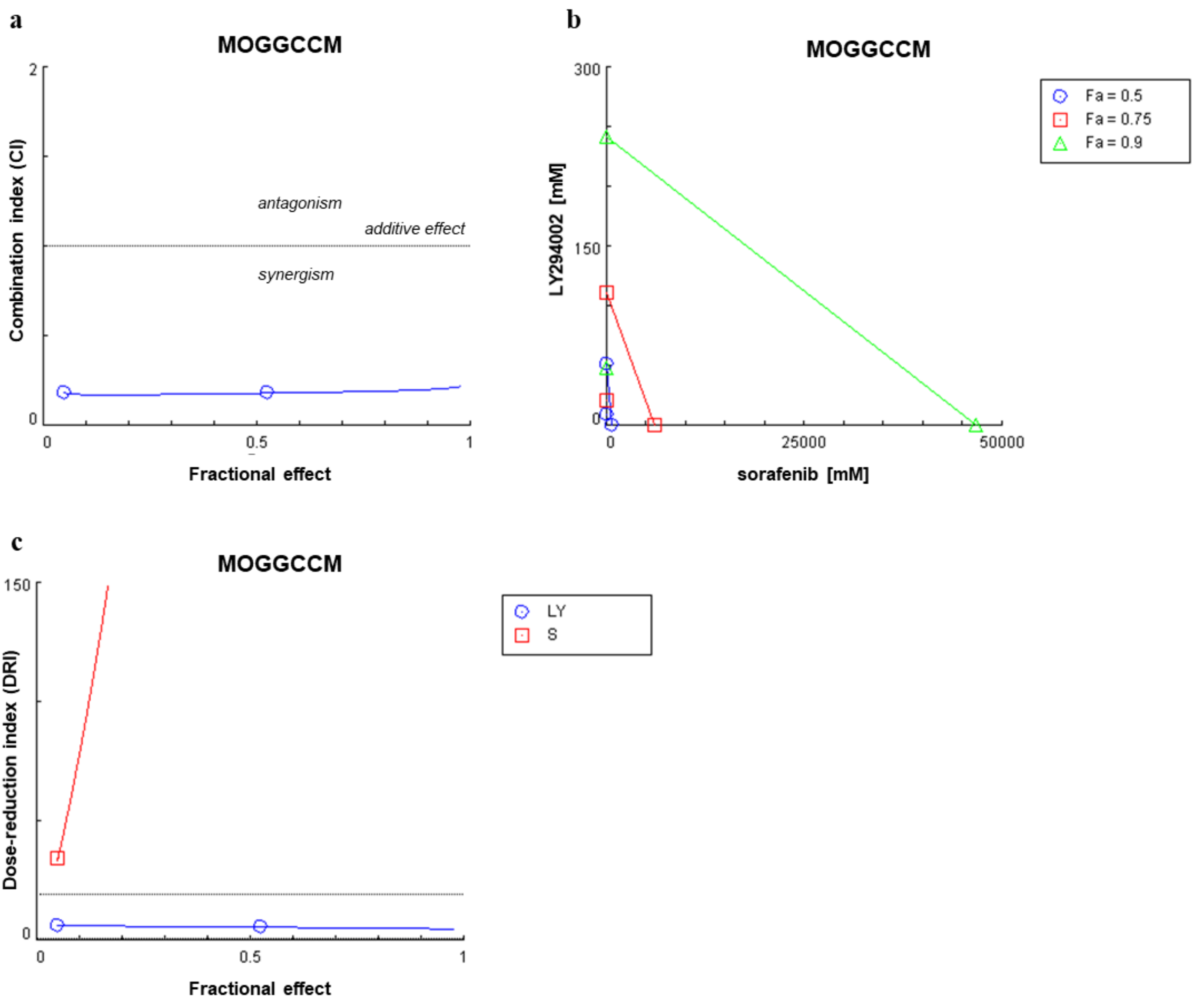

Fig. 5 A: LY294002 and sorafenib combination treatment in MOGGCCM cells. (a) Combination index (CI) plot: The combination index is plotted as a function of Fa (fractional effect). (b) Isobologram for combination: Classic isobologram at IC_{50}, IC_{75}, and IC_{90}. (c) the Fa-DRI (dose reduction index) plot (Chou-Martin plot). Combination index (CI) and dose reduction index (DRI) were calculated according to the method of Chou and Talalay using the Compusyn software. CI<1, CI=1 and CI>1 indicate synergistic effect, additive effect, and antagonistic effect, respectively. The DRI represents the fold reduction of LY294002 as a result of synergistic combination compared to the concentration of drug treatment alone needed to reach the same effect. The use of a combination of LY294002 and sorafenib in the MOGGCCM cells line resulted in synergism.

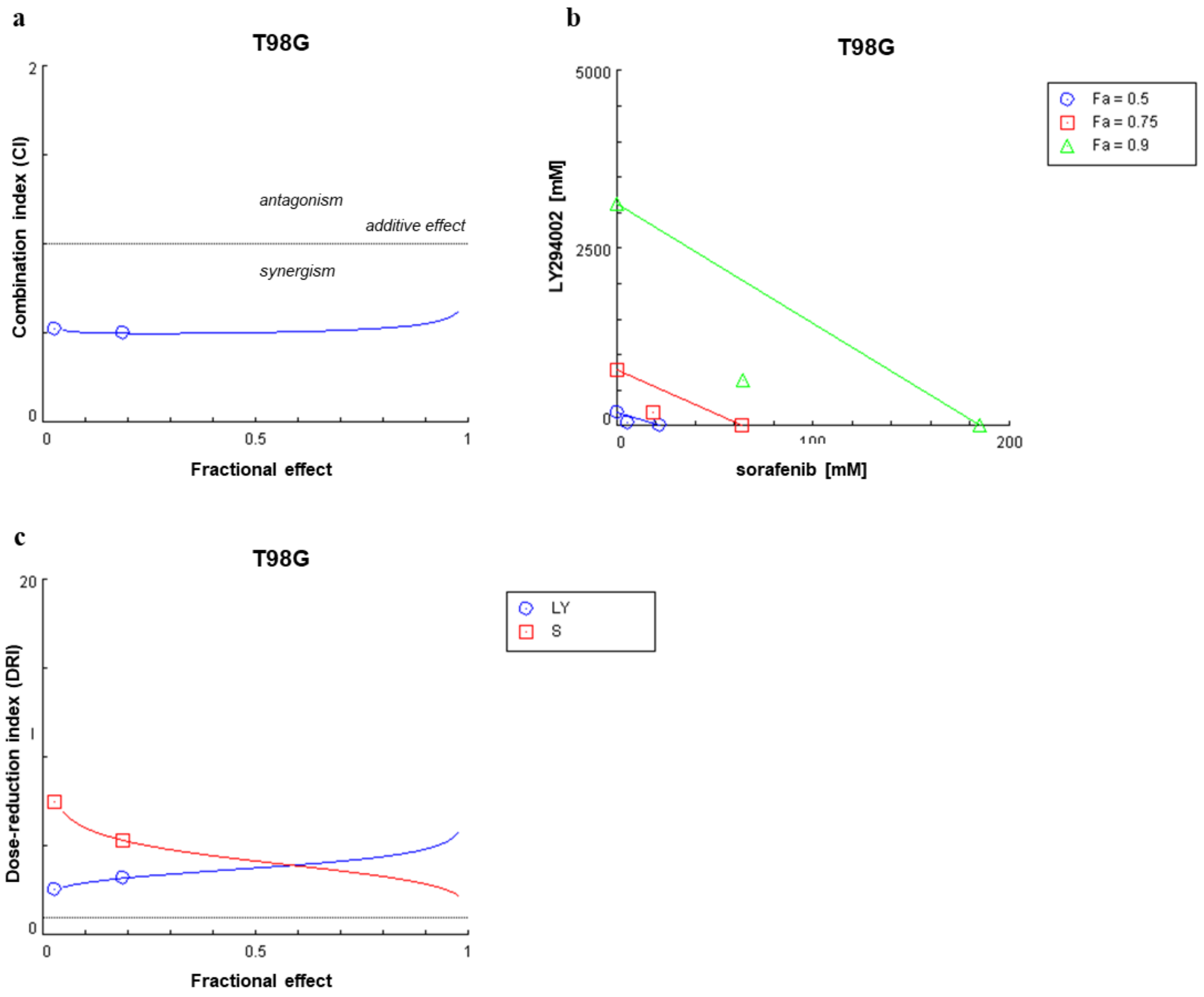

Fig. 6 LY294002 and sorafenib combination treatment in T98G cells. (a) Combination index (CI) plot: The combination index is plotted as a function of Fa (fractional effect). (b) Isobologram for combination: Classic isobologram at IC_{50}, IC_{75}, and IC_{90}. (c) the Fa-DRI (dose reduction index) plot (Chou-Martin plot). Combination index (CI) and dose reduction index (DRI) were calculated according to the method of Chou and Talalay using the Compusyn software. CI<1, CI=1 and CI>1 indicate synergistic effect, additive effect, and antagonistic effect, respectively. The DRI represents the fold reduction of TMZ as a result of synergistic combination compared to the concentration of drug treatment alone needed to reach the same effect. These results showed the efficacy of LY294002 to improve sorafenib-mediated cytotoxicity by reducing its concentration. Therefore, LY294002 in combination with sorafenib have a synergistic effect on the apoptosis induction of glioma cells.

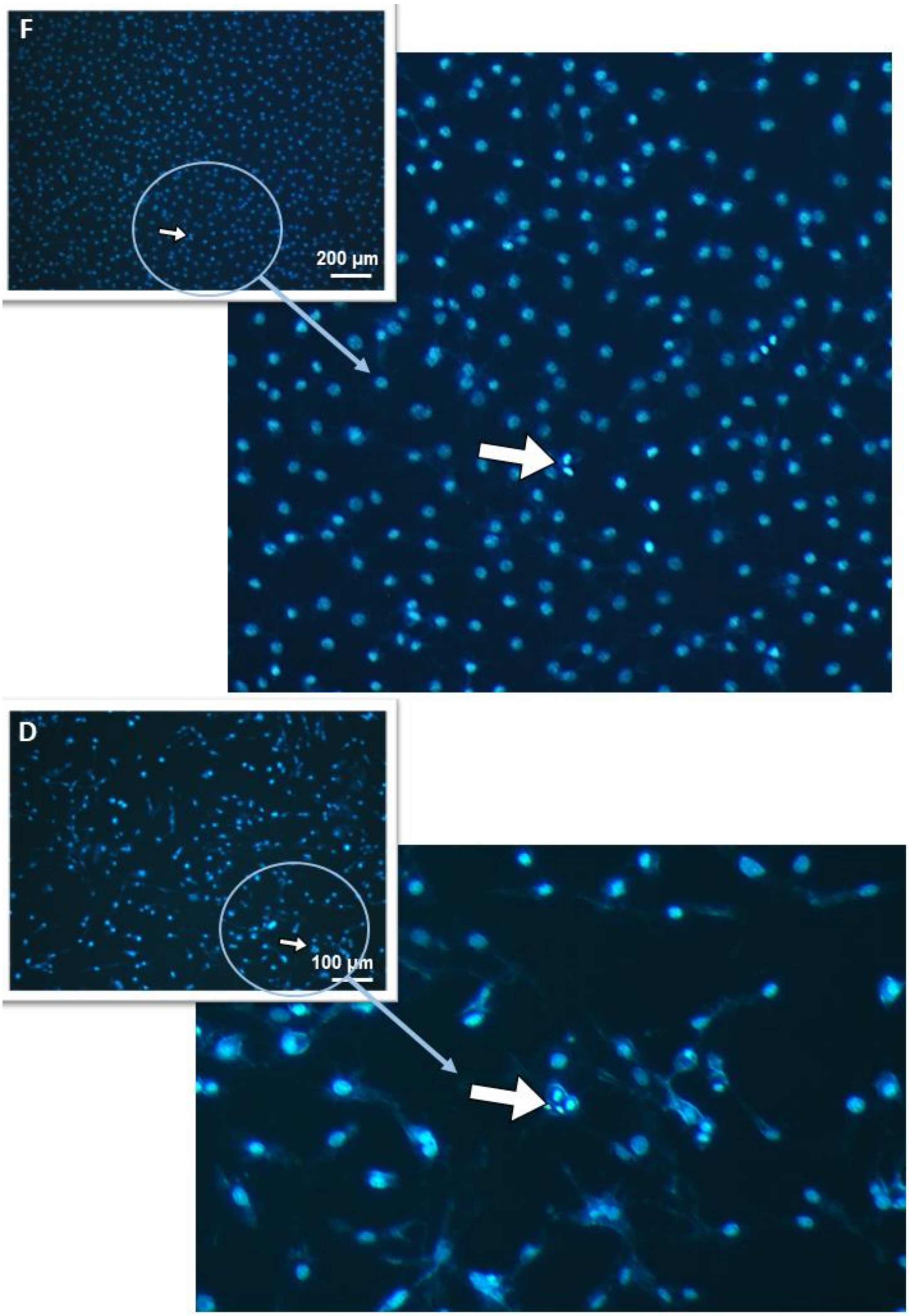

Fig. 7 Zoomed photos of normal (astrocytes and oligodendrocytes) treated cells for apoptotic bodies visualization increasement.
